# Blood Flow Restriction Training Improves Cardiac Structure and Diastolic Function in Runners with Exercise-Induced Hypertension

**DOI:** 10.3390/jcm14217795

**Published:** 2025-11-03

**Authors:** Young-Joo Kim, Jong-Young Lee, Choung-Hwa Park, Han-Soo Park

**Affiliations:** 1 School of Sports Science, Sungshin Women’s University, Seoul 02844, Republic of Korea; kyj87@sungshin.ac.kr; 2 Division of Cardiology, Department of Internal Medicine, Hallym University Sacred Heart Hospital, Anyang 14068, Republic of Korea; jyleeheart@naver.com; 3 Graduate School of Alternative Medicine, Kyonggi University, Seoul 02844, Republic of Korea; 4 Sports Medicine Laboratory, Korean National Sport University, Seoul 05541, Republic of Korea

**Keywords:** blood flow restriction training, exercise-induced hypertension, echocardiography, diastolic function, cardiac remodeling

## Abstract

**Background/Objectives**: Exercise-induced hypertension (EIH) in runners predisposes them to cardiovascular diseases, including myocardial hypertrophy, arrhythmias, and coronary artery disease. Blood flow restriction (BFR) training has been reported to exert non-pharmacological benefits in runners with EIH by improving blood pressure, myocardial workload, and cardiorespiratory fitness. The purpose of this study was to investigate whether changes in myocardial structure and function accompany these effects of BFR training in middle-aged runners with EIH. **Methods**: Participants who exhibited a maximal systolic blood pressure of ≥210 mmHg during an exercise stress test were assigned either to a BFR training group (BFRTg, *n* = 15) or to a control group without BFR training (non-BFRTg, *n* = 14). The BFRTg underwent a two-month BFR training program, performed twice per week for 20 min per session. Cardiac structure and function were evaluated before and after the intervention, and exercise stress test data were obtained from secondary sources of a previous study. **Results**: Compared with controls, the BFR group showed lower maximal exercise SBP, longer exercise duration, and higher VO_2max_. Echocardiography revealed reduced interventricular septal thickness and improved diastolic indices (higher E′/A′, lower E/E′), while systolic function remained unchanged. **Conclusions**: In conclusion, reductions in septal thickness and improvements in diastolic function induced by blood flow restriction training in runners with exercise-induced hypertension suggest a favorable cardiac adaptation, accompanied by concurrent improvements in exercise blood pressure and cardiorespiratory fitness.

## 1. Introduction

Regular physical activity is an important strategy for reducing cardiovascular and cerebrovascular events in patients with coronary artery disease, thereby lowering mortality [1] and improving quality of life [2]. However, despite these well-established benefits, excessive endurance exercise has been associated with a paradoxical elevation in blood pressure during exertion, known as exercise-induced hypertension (EIH). The prevalence of EIH is higher among runners who engage in strenuous endurance activities such as marathon running compared with the general population [3]. In these individuals, EIH is associated with an increased risk of arrhythmia, coronary artery disease, and myocardial hypertrophy [3,4,5].

In this study, EIH is diagnosed if systolic blood pressure measured during a standardized treadmill GXT (Bruce protocol) reaches ≥ 210 mmHg in men and ≥190 mmHg in women, with blood pressure assessed at rest, during each stage, and at maximal exertion [6]. This approach reflects the clinical definition of EIH as an excessive rise in blood pressure during maximal exercise, thereby imposing sustained hemodynamic stress that leads to maladaptive structural and functional changes in the myocardium and vasculature [7,8,9]. Clinically, these maladaptive changes may increase the risk of adverse cardiac events, including sudden cardiac death, particularly during endurance competition [9].

Although pharmacological approaches have been discussed in the context of managing exercise-induced hypertension, there are currently no established pharmacological recommendations or official guidelines for its treatment [10]. Because EIH typically occurs in otherwise healthy athletes, the development of safe, exercise-based interventions to normalize blood pressure responses during exertion represents an important unmet clinical need.

Blood flow restriction (BFR) training, which combines low- to moderate-intensity exercise with partial restriction of blood flow to the limbs, has gained increasing attention as a novel training modality [11]. This approach has been shown to enhance aerobic capacity [12], promote muscle hypertrophy and strength [13], and accelerate recovery following anterior cruciate ligament injury [14]. The transient ischemic and metabolic stress induced by BFR training enhances endothelial function and stimulates muscle growth [15]. However, most previous studies have examined only the acute or chronic effects of BFR training on resting blood pressure [16,17] or muscular outcomes, without investigating its potential influence on cardiac structure and function. Recent findings from our group demonstrated that BFR training reduced blood pressure during exercise, maximal systolic blood pressure, and myocardial workload in runners with EIH [18]. However, whether BFR training can induce favorable cardiac structural and functional adaptations in runners with EIH remains unclear.

Therefore, the present study aimed to determine whether the benefits of BFR training in runners with EIH—specifically, reductions in maximal systolic blood pressure and improvements in cardiorespiratory fitness—are accompanied by concomitant improvements in cardiac structure and function. We hypothesized that BFR training would reduce maximal exercise systolic blood pressure and promote favorable cardiac adaptation, characterized by decreased interventricular septal thickness and enhanced left ventricular diastolic function.

## 2. Materials and Methods

### 2.1. Participants and the Study Protocol

This study was a prospective quasi-experimental sub-study derived from the cohort previously reported by Kim et al. [18]. The same 8-week blood flow restriction (BFR) training protocol and graded exercise testing were used, and echocardiographic assessments were newly performed after the intervention to evaluate cardiac structural and functional adaptations. Participants voluntarily selected whether to join the BFR program (BFRT group) or not (non-BFRT group); hence, the design was non-randomized but prospective. Due to incomplete echocardiographic data, the final sample size differed slightly from the original cohort, although baseline and exercise test characteristics remained comparable.

Table 1 presents the baseline characteristics of the participants. Participants with EIH, defined as a maximal systolic blood pressure (SBPmax ≥ 210 mmHg) during a graded exercise test (GXT), were selected for inclusion. From this cohort, participants who voluntarily participated in a two-month BFR training program (20 min per session, twice per week) were assigned to the blood flow restriction training group (BFRTg, *n* = 15), while those who did not participate were assigned to the control group (non-BFRTg, *n* = 14). There were no significant intergroup differences in physical characteristics (age, height, weight, BMI) or training history (years of training experience, marathon personal best, number of completed marathons, exercise intensity, exercise frequency, and typical exercise duration). In the GXT, no significant differences were observed between groups in HRrest, HRmax, SBPrest, SBPmax, DBPrest, DBPmax, maximal arrival time (MAT), or maximal oxygen uptake (VO_2max_).

**Figure 1:**
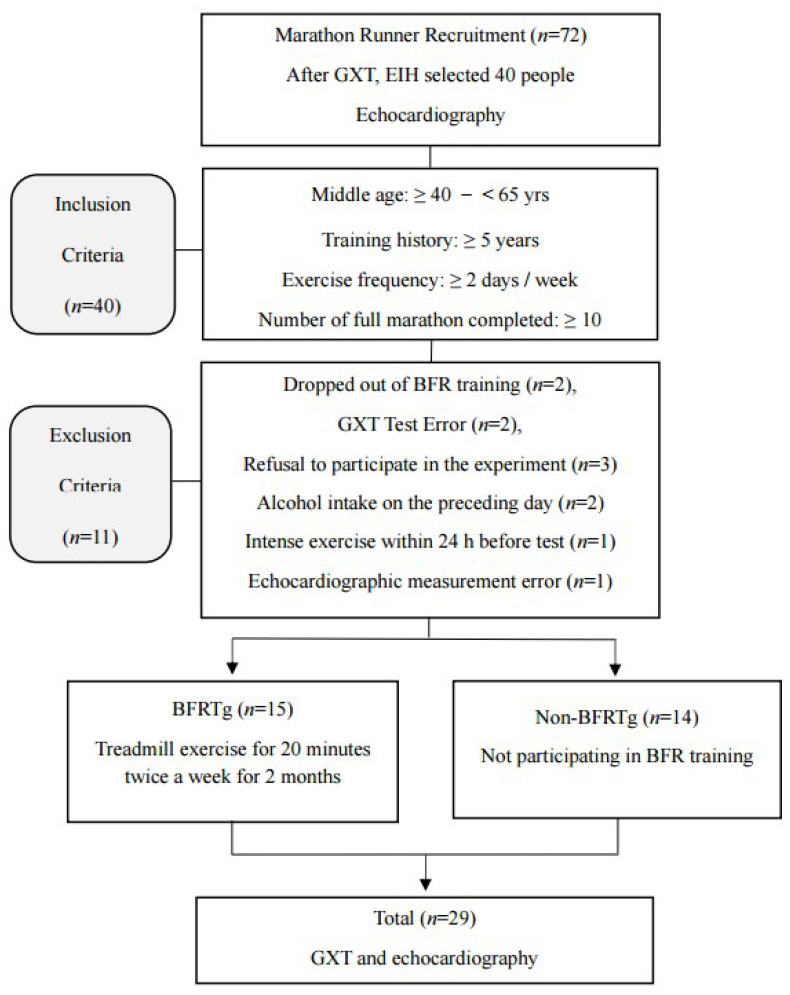
Flow chart of the study procedure. GXT, graded exercise testing; EIH, exercise induced hypertension; BFR, blood flow restriction; BFRTg, blood flow restriction training group.

The overall participant flow is illustrated in Figure 1. A total of 72 male long-distance runners were initially recruited and underwent graded exercise testing (GXT) to identify individuals with exercise-induced hypertension (EIH), defined as a maximal systolic blood pressure ≥ 210 mmHg in men according to established criteria [6]. Forty runners met the EIH criteria and were eligible for inclusion. During the intervention period, 11 participants were excluded for the following reasons: withdrawal from training (*n* = 2), GXT error (*n* = 2), refusal to continue (*n* = 3), alcohol intake within 24 h before testing (*n* = 2), intense exercise within 24 h before testing (*n* = 1), or echocardiographic measurement error (*n* = 1). Consequently, 29 participants completed both the training and echocardiographic assessments and were included in the final analysis (BFRTg, *n* = 15; non-BFRTg, *n* = 14). No a priori power analysis was performed because this study was an exploratory sub-analysis; however, the sample size was comparable to previous echocardiographic intervention trials in runners with EIH.

This study was conducted in accordance with the principles of the 1975 Declaration of Helsinki and was approved by the Institutional Review Board of the Korea National Sport University (approval no. 20230921-091).

### 2.2. Graded Exercise Test (GXT)

Participants underwent a GXT to evaluate hemodynamic responses and cardiorespiratory fitness. The GXT was conducted on a treadmill (T170DE, hp cosmos, Traunstein, Germany) using the Bruce protocol. Hemodynamic responses, including heart rate (HR), systolic blood pressure (SBP), and diastolic blood pressure (DBP), were measured at rest, during each stage, and at maximal exertion. Assessments were performed using a respiratory gas analyzer (Quark CPET, COSMED, Rome, Italy), an automated blood pressure monitor (Tango+, SunTech Medical, Morrisville, NC, USA), and a heart rate and electrocardiogram (ECG) system (CH2000, Cambridge Heart, Bedford, MA, USA).

VO_2max_ was recorded as an indicator of cardiorespiratory fitness, and rating of perceived exertion (RPE) was assessed using the Borg scale. To ensure accuracy, blood pressure was also measured manually by a trained operator using headphones, with a high-performance microphone positioned over the brachial artery. All GXT procedures followed the American College of Cardiology/American Heart Association (ACC/AHA) guidelines [19].

### 2.3. Echocardiography Test

Echocardiographic examinations were performed by a veteran cardiac sonographer at Korea University Guro Hospital. The assessments were conducted using an Alpha6 Hitachi Aloka ultrasound system (Tokyo, Japan) with a 2–5 MHz transducer. To ensure accurate measurements, participants rested in a seated position for five minutes, after which their blood pressure and heart rate were recorded. Participants were assessed in the left lateral decubitus position, and the following were measured using M-mode echocardiography in accordance with the American Society of Echocardiography/European Association of Cardiovascular Imaging (ASE/EACVI) guidelines [20]: left atrial diameter (LAD), left ventricular internal dimension at end-diastole (LVIDd), left ventricular internal dimension at end-systole (LVIDs), left ventricular posterior wall thickness at end-diastole (LVPWd), interventricular septum thickness at end-diastole (IVSd), left ventricular mass (LVM), left ventricular mass index (LVMI), left ventricular end-diastolic volume (LVEDV), left ventricular end-systolic volume (LVESV), left ventricular stroke volume (LVSV), cardiac output (CO), cardiac output index (COI), left ventricular ejection fraction (LVEF), and left ventricular fractional shortening (LVFS). Indexed values were calculated by dividing by the body surface area (BSA). To assess diastolic function, pulsed-wave tissue Doppler imaging was used to measure peak early (E) and late (A) diastolic filling velocities, early (E′) and late (A′) diastolic mitral annular velocities, and deceleration time (DT), as well as the ratios E/A, E′/A′, and E/E′.

### 2.4. Blood Flow Restriction Exercise Method

The exercise intensity for all participants was set at 40–60% of Heart Rate Reserve (HRR), calculated using the Karvonen formula with resting and maximal heart rates obtained from the GXT. HRR values were fixed from baseline GXT data and progressively increased from approximately 40% to 60% over the 8-week intervention period to ensure consistent and gradual intensity progression. Exercise was performed on a treadmill while wearing a BFR device, with speed and grade adjusted to achieve the target intensity. The training program consisted of 20 min sessions conducted twice weekly for two months.

During the first week, which served as a BFR adaptation period, treadmill exercise was performed at 50% HRR. Cuffs were applied to the proximal thighs, and the Cyclic mode (Low SKU, 150–220 mmHg) was employed. This protocol consisted of a repeating cycle of a 30 s pressure application followed by a 5 s release. The pressure began at 150 mmHg and was increased by 10 mmHg in each subsequent cycle until 220 mmHg was reached, with this entire procedure applied for 20 min.

In the second week, the Cyclic mode (Medium SKU, 230–300 mmHg) was used. For the first 10 min, the cyclic protocol (30 s application, 5 s release) was performed with incremental pressure increases of 10 mmHg, beginning at 230 mmHg and continuing until 300 mmHg was reached. The protocol was then switched to the Constant mode, in which pressure was sustained at 250 mmHg without release for the remaining 10 min.

From the third to the eighth week, the protocol was modified. After performing the initial pressure ramp-up using the Cyclic mode only once, participants exercised under continuous blood flow restriction for 15 min while maintaining an intensity of 60% HRR. The detailed protocol of the BFR training is summarized in Table 2.

### 2.5. Statistical Analysis

All analyses were performed using SPSS Statistics version 21 (IBM Corp., Armonk, NY, USA), and data are presented as mean ± standard deviation. Baseline differences between the BFRTg and non-BFRTg were examined using independent-samples *t*-tests or Mann–Whitney *U* tests, depending on data normality (Shapiro–Wilk test). Intervention effects were analyzed using two-way repeated-measures ANOVA to assess the main effects of time (pre vs. post), group (BFRTg vs. non-BFRTg), and their interaction. When the assumption of sphericity was violated (Mauchly’s test), Greenhouse–Geisser correction was applied. A significant interaction (*p* < 0.05) was interpreted as a difference in the magnitude or direction of change over time between groups. Post hoc comparisons were performed using paired or independent *t*-tests (for normal data) or Wilcoxon and Mann–Whitney tests (for non-normal data). Missing data were handled by listwise deletion. Effect sizes were calculated as partial eta squared (*η*^2^) for ANOVA and Cohen’s *d* for *t*-tests. Statistical significance was set at *p* < 0.05 (two-tailed).

## 3. Results

### 3.1. Graded Exercise Test (GXT) Outcomes

A significant time × group interaction was observed for maximal systolic blood pressure (SBP_max_, *p* < 0.001, two-way repeated-measures ANOVA). Post hoc analysis revealed a marked decrease in SBP_max_ in the BFRT group (222.8 ± 7.3 → 179.7 ± 17.4 mmHg; *p* < 0.001, paired *t*-test), whereas no significant change occurred in the control group. Exercise duration and VO_2max_ also showed significant time × group interactions (*p* < 0.05), indicating improved exercise capacity following BFR training.

Table 3 summarizes the graded exercise test results, showing concurrent reductions in exercise systolic blood pressure and improvements in exercise performance variables in the BFRT group compared with controls.

### 3.2. Echocardiography Outcomes

A significant time × group interaction was identified for interventricular septal thickness (LVIVSd, *p* = 0.009). The BFRT group demonstrated a reduction in LVIVSd (0.96 ± 0.09 → 0.92 ± 0.07 cm), while the control group showed a slight increase (0.95 ± 0.11 → 0.97 ± 0.11 cm). Other echocardiographic indices, including left ventricular dimensions, posterior wall thickness, and systolic function (LVEF, LVFS), exhibited no significant interaction (*p* > 0.05).

**Figure 2:**
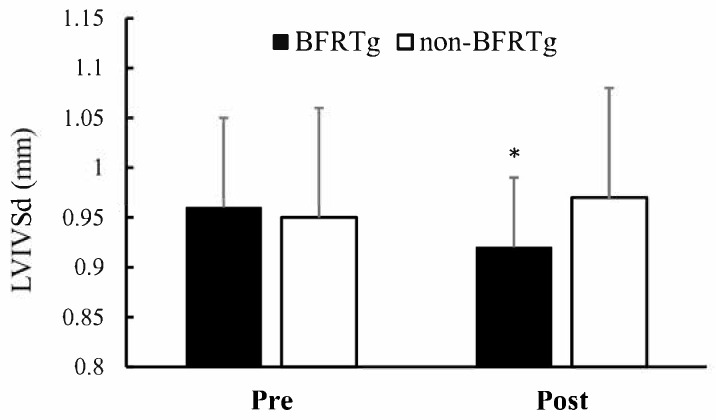
Pre- and Post-BFR Training Changes in LVIVSd. LVIVSd, left ventricular Interventricular Septal thickness in diastole; BFRTg, blood flow restriction training group. * *p* < 0.05, pre vs. post.

Figure 2 visually illustrates the septal thickness reduction in the BFRT group, while Table 4 numerically summarizes these echocardiographic findings, confirming structural adaptation without any decline in systolic performance.

Significant time × group interactions were observed for both the E′/A′ ratio (*p* = 0.021) and E/E′ ratio (*p* = 0.005). The BFRT group showed improved diastolic function, reflected by an increase in E′/A′ (1.08 ± 0.15 → 1.21 ± 0.13) and a decrease in E/E′ (9.6 ± 2.1 → 8.3 ± 1.9), whereas no significant changes were found in the control group.

**Figure 3:**
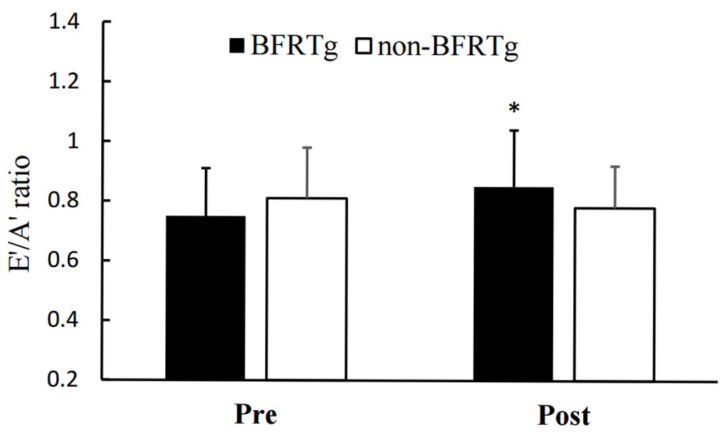
Pre- and Post-BFR Training Changes in E′/A′. E′, early diastolic annulus velocity; A′, late diastolic annulus velocity; BFRTg, blood flow restriction group. * *p* < 0.05, pre vs. post.

**Figure 4:**
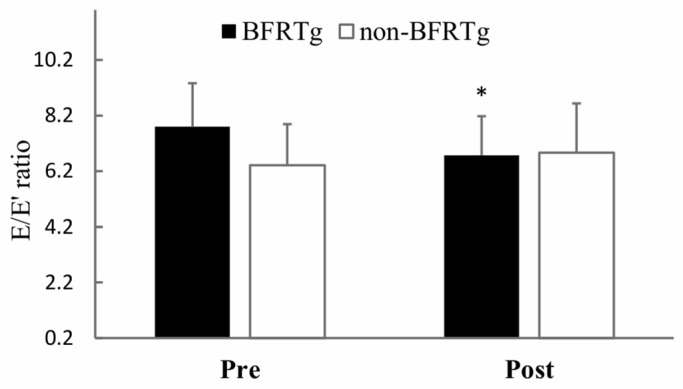
Pre- and Post-BFR Training Changes in E/E′ ratio. E, peak velocity of early filling; E′, early diastolic annulus velocity; BFRTg, blood flow restriction group. * *p* < 0.05, pre vs. post.

Figure 3 and Figure 4 visually illustrate the diastolic improvements, and Table 5 numerically presents the corresponding data, indicating enhanced left ventricular relaxation while maintaining normal systolic function.

## 4. Discussion

The present study evaluated the newly assessed cardiac structural and functional adaptations (echocardiography) following a two-month BFR training program in runners with EIH. This investigation extends our previous work on this same cohort [18], which established the foundational hemodynamic and cardiorespiratory fitness data from the GXT. As detailed in that prior report, the GXT findings—reductions in maximal systolic blood pressure, prolonged exercise duration, and improved cardiorespiratory fitness (Table 3)—are consistent with previous studies and serve as the essential reference data for interpreting the novel echocardiographic results presented here.

Previous studies on the effects of BFR training have primarily focused on reductions in resting blood pressure [16,21] or demonstrated significant decreases after resistance exercise [22]. In addition, BFR training has been associated with enhanced endothelial function, vasodilation [23,24], and reduced arterial stiffness [25,26,27], providing a theoretical mechanism for attenuating excessive blood pressure elevation. Our previously reported GXT results [18] are in line with these mechanisms and provide the critical context for the new echocardiographic findings of the current study.

The increase in VO_2max_ is significant given that all participants were trained runners who regularly engaged in structured exercise. A meta-analysis by Gao et al. [26] involving 270 participants reported that aerobic exercise combined with BFR resulted in greater improvements in VO_2max_ compared to exercise without BFR. The present results are consistent with this evidence, suggesting that BFR training can induce additional gains in cardiorespiratory fitness even among runners with EIH who already possess a high level of training status.

Echocardiographic assessments in this study suggest, for the first time, that BFR training induces adaptive changes in cardiac structure and function in runners with EIH. This is the first study to show echocardiographic evidence of favorable reverse remodeling following BFR training in endurance-trained runners with EIH, clearly distinguishing it from prior investigations that primarily examined resting blood pressure or peripheral adaptations.

Compared with the non-BFRTg, the BFRTg exhibited a significant reduction in left ventricular interventricular septal thickness (LVIVSd). This finding aligns with those of Kokkinos et al. [28] and Hinderliter et al. [29], who reported that regular exercise in hypertensive patients reduces blood pressure while also improving left ventricular hypertrophy (LVH) and left ventricular mass (LVM). Although participants in the present study did not meet the diagnostic threshold for LVH (LVMI ≥ 115 g/m^2^) [20], the observed decrease in septal thickness following BFR training may be interpreted as a positive structural adaptation, likely mediated by reductions in maximal exercise blood pressure and associated myocardial workload. Importantly, the selective reduction in IVSd without concurrent changes in LV mass can be regarded as an early signal of reverse remodeling. Prior evidence indicates that in intensive blood pressure lowering trials such as SPRINT-HEART, regression of septal wall thickness can emerge within weeks to months of effective hemodynamic unloading [30], whereas in long-term ACE inhibitor cohort studies, more global LV mass regression typically requires several months of sustained intervention, is often observed within the first year, and continues to progress with extended follow-up [31,32].

Furthermore, to our knowledge, this is the first study to demonstrate improvements in left ventricular diastolic function following BFR training in this population. While no intergroup differences were found for E, A, E/A, or DT, significant improvements were observed in the E′/A′ and E/E′ ratios in the BFRTg. Notably, the E/E′ ratio is a critical diagnostic and prognostic marker for heart failure [33,34,35], and is widely used to predict morbidity and mortality in patients with hypertension and cardiovascular disease [36,37,38]. Evidence from meta-analyses showing that exercise training significantly reduces the E/E′ ratio in patients with heart failure [39,40] supports the interpretation that BFR training may also contribute to enhanced left ventricular diastolic function.

The improvements in E′/A′ and E/E′ observed here are clinically meaningful, as they reflect reductions in filling pressures and improved diastolic relaxation, even in highly trained endurance runners.

What is particularly noteworthy is that BFR training reduced excessive elevations in blood pressure and improved cardiorespiratory fitness even in EIH runners accustomed to high-intensity exercise, such as veteran marathoners. For this population, it is generally difficult to achieve further reductions in blood pressure through exercise alone. In contrast, the unique stimuli provided by BFR, namely metabolic and transient ischemic stress, are known to enhance endothelial function and reduce arterial stiffness [16,25,26,27,28]. However, the observed increases in cardiorespiratory fitness, suppression of excessive blood pressure surges, reductions in septal thickness, and improvements in diastolic function demonstrate the potential of BFR training as a non-pharmacological alternative therapy.

Future studies should investigate whether increasing the frequency and duration of BFR training can elicit beneficial changes in other structural indices, such as left ventricular (LV) mass. Furthermore, large-scale, long-term follow-up studies are warranted to determine whether these effects can be sustained and to establish clinical guidelines based on this evidence. This study has the following limitations.

First, the relatively small sample size limits the generalizability of our findings. Nevertheless, the homogeneity of participants—all runners with exercise-induced hypertension—provides strong internal validity.

Second, participants were not randomly assigned but self-selected whether to participate in the blood flow restriction training (BFRT) program or not. Although this design introduces the possibility of self-selection bias, baseline characteristics were comparable between groups, suggesting that its influence on the study outcomes was likely minimal.

Third, the intervention period was limited to 8 weeks with twice-weekly sessions. This relatively short duration may not have been sufficient to elicit broader structural remodeling such as reductions in LV mass or posterior wall thickness. Future studies with longer and more frequent interventions are needed.

Fourth, all participants were experienced marathon runners. Thus, the findings may not be generalizable to novice runners, recreational athletes, or the general hypertensive population. However, the fact that favorable adaptations were observed even in highly trained athletes strengthens the clinical relevance of BFR training.

Fifth, mechanistic biomarkers such as nitric oxide, endothelin-1, VEGF, or indices of arterial stiffness were not measured. Including such biomarkers in future studies would help clarify the physiological pathways underlying the observed cardiac adaptations.

Sixth, this study was a secondary analysis of an existing cohort, and the exercise test results were largely consistent with previously reported findings. Therefore, the novel contribution lies primarily in the echocardiographic evidence of cardiac structural and functional improvements. While this limits originality, it also underscores the robustness and reproducibility of the findings.

Seventh, the absence of long-term follow-up prevents conclusions about the persistence of these benefits. Longitudinal studies are warranted to determine whether the observed improvements are sustained and translate into reduced cardiovascular morbidity.

Finally, echocardiographic assessments were not performed under blinded conditions, which may have introduced potential observer bias despite adherence to standardized measurement protocols.

## 5. Conclusions

BFR training not only mitigated excessive blood pressure elevation during graded exercise testing in runners with exercise-induced hypertension (EIH) but also improved cardiorespiratory fitness. Echocardiographic evaluations further demonstrated reductions in interventricular septal thickness and enhancements in diastolic function. These results indicate that BFR training may represent a promising non-pharmacological therapeutic strategy capable of eliciting favorable structural and functional cardiac adaptations, even in runners with EIH.

## Figures and Tables

**Table 1:** General characteristics, exercise data, and GXT data.

Variable	BFRTg (*n* = 15)	Non-BFRTg (*n* = 14)	*p*-Value
General characteristics			
Age (years)	57.1 ± 5.6	57.1 ± 7.4	0.997
Height (cm)	172.0 ± 7.7	170.3 ± 3.6	0.462
Weight (kg)	68.4 ± 7.2	63.3 ± 16.3	0.280
BMI (m^2^/kg)	23.1 ± 2.2	21.8 ± 5.5	0.409
Disease	9 (60%)	6 (42.8%)	0.580
Hypertension	5 (33.3%)	2 (14.3%)	0.231
Dyslipidemia	3 (20.0%)	2 (14.3%)	0.684
Diabetes	1 (6.7%)	1 (7.1%)	0.960
Diabetes + Hypertension	0 (0.0%)	1 (7.1%)	0.292
Exercise data			
Exercise history (years)	15.8 ± 6.2	17.5 ± 6.3	0.474
Marathon time (min)	234.6 ± 39.0	234.6 ± 39.0	0.833
Marathon completed (numbers)	73.6 ± 54.0	54.7 ± 39.5	0.296
Exercise intensity (Borg’s scale)	14.3 ± 1.4	14.8 ± 0.9	0.263
Exercise frequency (weeks)	3.7 ± 1.7	3.8 ± 1.5	0.841
Exercise time (min/day)	74.6 ± 22.6	87.1 ± 30.0	0.219
GXT			
HRrest (beats/min)	60.0 ± 8.0	60.2 ± 7.0	0.939
HRmax (beats/min)	163.5 ± 14.4	166.9 ± 9.2	0.462
SBPrest (mmHg)	129.0 ± 12.2	123.9 ± 9.6	0.229
SBPmax (mmHg)	222.8 ± 7.3	218.2 ± 5.5	0.068
DBPrest (mmHg)	83.3 ± 9.7	78.2 ± 5.4	0.095
DBPmax (mmHg)	94.8 ± 7.3	94.0 ± 9.9	0.795
TET (s)	761.3 ± 130.4	806.4 ± 96.5	0.302
VO_2max_ (mL/kg/min)	46.0 ± 9.6	47.2 ± 6.1	0.700

Values are presented as mean ± standard deviation. BFRTg, blood flow restriction group; BMI, body mass index; GXT, graded exercise test; HR, heart rate; SBP, systolic blood pressure; DBP, diastolic blood pressure; TET, total exercise time.

**Table 2:** Summary of the 8-Week Blood Flow Restriction (BFR) Training Protocol.

Week	Target Intensity (%HRR)	Frequency & Duration	Mode and Pressure Details	Description
Week 1	50% HRR	Twice weekly, 20 min	Cyclic mode (low pressure: 150–220 mmHg); 30 s inflation → 5 s release, with +10 mmHg increments per cycle	Adaptation phase to gradually expose participants to BFR stimulus
Week 2	50% HRR	Twice weekly, 20 min	Stage 1: Cyclic mode (230–300 mmHg) for 10 min with gradual pressure increase (+10 mmHg per cycle) Stage 2: Constant mode (250 mmHg) for 10 min of sustained restriction	Transition phase introducing moderate pressure with partial constant loading
Weeks 3–8	60% HRR	Twice weekly, 20 min	Initial phase: Cyclic mode (230–300 mmHg) for 5 min with gradual pressure increments, followed by Continuous phase: Constant mode (250 mmHg) for 15 min of sustained restriction	Continuous BFR treadmill exercise at moderate intensity, promoting progressive load adaptation

**Table 3:** Change in graded exercise tests before and after BFR training.

Variable	Group	Pre	Post	*p*-Value
HR_rest_ (beats/min)	BFRTg	60.0 ± 8.0	62.2 ± 10.7	T: 0.187 G: 0.870 I: 0.875
	non-BFRTg	60.2 ± 7.0	63.0 ± 12.2	
SBP_rest_ (mmHg)	BFRTg	129.0 ± 12.2	119.5 ± 9.2 *	T: 0.027 G: 0.100 I: 0.770
	non-BFRTg	123.9 ± 9.6	122.5 ± 14.1	
DBP_rest_ (mmHg)	BFRTg	83.3 ± 9.7	84.7 ± 8.0	T: 0.202 G: 0.360 I: 0.084
	non-BFRTg	78.2 ± 5.4	85.7 ± 5.8 *	
HR_max_ (beats/min)	BFRTg	163.5 ± 14.4	161.1 ± 17.9	T: 0.882 G: 0.243 I: 0.317
	non-BFRTg	166.9 ± 9.2	168.7 ± 10.2	
SBP_max_ (mmHg)	BFRTg	222.8 ± 7.3	179.7 ± 17.4 * §	T: <0.001 G: <0.001 I: <0.001
	non-BFRTg	218.2 ± 5.5	211.4 ± 12.9	
DBP_max_ (mmHg)	BFRTg	94.8 ± 7.3	88.6 ± 8.7 *	T: 0.004 G: 0.838 I: 0.358
	non-BFRTg	94.0 ± 9.9	90.7 ± 10.3	
TET (sec)	BFRTg	761.3 ± 130.4	806.0 ± 134.7 *	T: 0.306 G: 0.714 I: 0.046
	non-BFRTg	806.4 ± 96.5	791.4 ± 99.4	
VO_2max_ (ml/kg/min)	BFRTg	46.0 ± 9.6	49.7 ± 8.4 *	T: 0.327 G: 0.544 I: <0.001
	non-BFRTg	47.2 ± 6.1	44.9 ± 8.1 *	

Values are presented as mean ± standard deviation, BMI, body mass index; HR, heart rate; SBP, systolic blood pressure; DBP, diastolic blood pressure; TET, total exercise time; T, time; G, group; I, interaction (time × group); *: significant difference in post for pre at *p* < 0.05; §: significant difference between BFRTg and non-BFRTg at *p* < 0.05.

**Table 4:** Pre- and Post-BFR Training Alterations in Cardiac Morphology and Systolic Function.

Variable	Group	Pre	Post	*p*-Value
LAD (cm)	BFRTg	3.6 ± 0.3	3.6 ± 0.2	T: 0.608 G: 0.116 I: 0.332
	Non-BFRTg	3.4 ± 0.4	3.4 ± 0.3	
LVIDd (cm)	BFRTg	4.7 ± 0.2	4.8 ± 0.2	T: 0.193 G: 0.595 I: 0.548
	Non-BFRTg	4.8 ± 0.4	4.9 ± 0.4	
LVIDs (cm)	BFRTg	3.0 ± 0.1	3.0 ± 0.1	T: 0.027 G: 0.100 I: 0.770
	Non-BFRTg	3.1 ± 0.3	3.1 ± 0.3	
LVPWd (mm)	BFRTg	0.92 ± 0.10	0.92 ± 0.12	T: 0.882 G: 0.243 I: 0.317
	Non-BFRTg	0.91 ± 0.14	0.92 ± 0.12	
LVIVSd (mm)	BFRTg	0.96 ± 0.09	0.92 ± 0.07	T: 0.195 G: 0.629 I: 0.009
	Non-BFRTg	0.95 ± 0.11	0.97 ± 0.11	
LVM (g)	BFRTg	180.8 ± 25.4	181.4 ± 30.8	T: 0.345 G: 0.515 I: 0.426
	Non-BFRTg	188.0 ± 53.6	194.2 ± 52.5	
LVMI (g/m^2^)	BFRTg	100.8 ± 17.2	100.6 ± 18.8	T: 0.413 G: 0.506 I: 0.373
	Non-BFRTg	104.9 ± 28.5	108.0 ± 27.8	
LVEDV (mL)	BFRTg	107.0 ± 11.3	108.6 ± 12.7	T: 0.170 G: 0.434 I: 0.572
	Non-BFRTg	111.6 ± 24.6	115.4 ± 26.9	
LVESV (mL)	BFRTg	37.4 ± 5.3	36.8 ± 4.8	T: 0.440 G: 0.441 I: 0.979
	Non-BFRTg	39.9 ± 12.2	39.2 ± 10.4	
LVSV (mL)	BFRTg	69.5 ± 8.9	72.0 ± 9.6	T: 0.049 G: 0.511 I: 0.532
	Non-BFRTg	71.4 ± 14.0	76.0 ± 17.6	
LVCO (L/min)	BFRTg	3.9 ± 0.8	4.0 ± 0.4	T: 0.291 G: 0.601 I: 0.493
	Non-BFRG	3.9 ± 0.8	4.3 ± 1.6	
LVEF (%)	BFRG	65.0 ± 4.1	66.1 ± 3.0	T: 0.054 G: 0.811 I: 0.694
	Non-BFRG	64.4 ± 4.3	66.1 ± 3.4	
LVFS (%)	BFRG	35.6 ± 3.0	36.5 ± 2.3	T: 0.059 G: 0.854 I: 0.694
	Non-BFRG	35.3 ± 3.1	36.5 ± 2.5	

Values are presented as mean ± standard deviation. LAD, left atrial diameter; LVIDd, left ventricular internal dimension end-diastolic; LVIDs, left ventricular internal dimension end-systolic; LVPWd, left ventricular posterior wall thickness end-diastolic; LVIVSd, left ventricular interventricular septum thickness end-diastolic; LVM, left ventricular mass; LVMI, left ventricular mass index; LVEDV, left ventricular end-diastolic volume; LVESV, left ventricular end-systolic volume; LVSV, left ventricular stroke volume; CO, cardiac output; COI, cardiac output index; LVEF, left ventricular ejection fraction; LVFS, left ventricular fractional shortening; T, time; G, group; I, interaction (time × group).

**Table 5:** Pre- and Post-BFR Training Alterations in Left Ventricular Diastolic Function.

Variable	Group	Pre	Post	*p*-Value
E (cm/s)	BFRG	68.4 ± 17.4	64.7 ± 13.4	T: 0.751 G: 0.231 I: 0.229
	Non-BFRG	59.1 ± 16.2	61.2 ± 14.0	
A (cm/s)	BFRG	62.2 ± 9.9	62.5 ± 10.8	T: 0.896 G: 0.267 I: 0.925
	Non-BFRG	58.1 ± 11.4	58.2 ± 10.3	
E/A	BFRG	1.12 ± 0.34	1.05 ± 0.24	T: 0.657 G: 0.862 I: 0.372
	Non-BFRG	1.05 ± 0.38	1.07 ± 0.29	
E′ (cm/s)	BFRG	8.7 ± 1.6	9.6 ± 1.6	T: 0.135 G: 0.946 I: 0.061
	Non-BFRG	9.2 ± 1.7	9.1 ± 1.8	
A′ (cm/s)	BFRG	11.8 ± 1.7	11.5 ± 0.9	T: 0.942 G: 0.983 I: 0.396
	Non-BFRG	11.5 ± 1.3	11.7 ± 1.8	
E′/A′ ratio	BFRG	0.75 ± 0.16	0.85 ± 0.19	T: 0.225 G: 0.902 I: 0.021
	Non-BFRG	0.81 ± 0.17	0.78 ± 0.14	
E/E′ ratio	BFRG	7.80 ± 1.56 §	6.77 ± 1.41	T: 0.240 G: 0.232 I: 0.005
	Non-BFRG	6.42 ± 1.47	6.87 ± 1.77	
DT (ms)	BFRG	0.18 ± 0.02	0.17 ± 0.01	T: 0.211 G: 0.101 I: 0.773
	Non-BFRG	0.19 ± 0.03	0.19 ± 0.01	

Values are presented as mean ± standard deviation. BFR, blood flow restriction; E, peak velocity of early filling; A, peak velocity of atrial filling; E′, Early diastolic annulus velocity; A′, late diastolic annulus velocity; DT, deceleration time; T, time; G, group; I, interaction (time × group). §, *p* < 0.05, between-group difference.

## Data Availability

The data presented in this study are available on request from the corresponding authors. The data are not publicly available due to privacy and ethical restrictions.

## Abbreviations

The following abbreviations are used in this manuscript:

ACC	American College of Cardiology
AHA	American Heart Association
ASE	American Society of Echocardiography
BFR	Blood Flow Restriction
BFRTg	Blood Flow Restriction Training group
BMI	Body Mass Index
BSA	Body Surface Area
CO	Cardiac Output
COI	Cardiac Output Index
DBP	Diastolic Blood Pressure
DBPmax	Maximal Diastolic Blood Pressure
DBPrest	Resting Diastolic Blood Pressure
DT	Deceleration Time
E	Peak early diastolic filling velocity
E′	Early diastolic mitral annular velocity
E/E′	Ratio of E to E′
E′/A′	Ratio of early to late diastolic mitral annular velocity
EIH	Exercise-Induced Hypertension
ECG	Electrocardiogram
GXT	Graded Exercise Test
HR	Heart Rate
HRmax	Maximal Heart Rate
HRrest	Resting Heart Rate
HRR	Heart Rate Reserve
IVSd	Interventricular Septum thickness at end-diastole
LAD	Left Atrial Diameter
LV	Left Ventricle/Left Ventricular
LVCO	Left Ventricular Cardiac Output
LVEDV	Left Ventricular End-Diastolic Volume
LVEF	Left Ventricular Ejection Fraction
LVESV	Left Ventricular End-Systolic Volume
LVFS	Left Ventricular Fractional Shortening
LVIDd	Left Ventricular Internal Dimension at end-diastole
LVIDs	Left Ventricular Internal Dimension at end-systole
LVIVSd	Left Ventricular Interventricular Septum thickness at end-diastole
LVM	Left Ventricular Mass
LVMI	Left Ventricular Mass Index
LVSV	Left Ventricular Stroke Volume
LVPWd	Left Ventricular Posterior Wall thickness at end-diastole
RPE	Rating of Perceived Exertion
SBP	Systolic Blood Pressure
SBP_max_	Maximal Systolic Blood Pressure
SBP_rest_	Resting Systolic Blood Pressure
TET	Total Exercise Time
VO_2max_	Maximal Oxygen Uptake
